# Numerical Investigation on Guided Waves Dispersion and Scattering Phenomena in Stiffened Panels

**DOI:** 10.3390/ma15010074

**Published:** 2021-12-23

**Authors:** Alessandro De Luca, Donato Perfetto, Giuseppe Lamanna, Antonio Aversano, Francesco Caputo

**Affiliations:** Department of Engineering, University of Campania “L. Vanvitelli”, Via Roma 29, 81031 Aversa, Italy; alessandro.deluca@unicampania.it (A.D.L.); giuseppe.lamanna@unicampania.it (G.L.); antonio.aversano@unicampania.it (A.A.); francesco.caputo@unicampania.it (F.C.)

**Keywords:** guided waves, structural health monitoring (SHM), finite element analysis (FEA), metals, composites, stiffener, reflection, transmission, mode conversion

## Abstract

The aim of this work is to propose a numerical methodology based on the finite element (FE) method to investigate the dispersive behavior of guided waves transmitted, converted, and reflected by reinforced aluminum and composite structures, highlighting their differences. The dispersion curves of such modes can help designers in improving the damage detection sensitivity of Lamb wave based structural health monitoring (SHM) systems. A preliminary phase has been carried out to assess the reliability of the modelling technique. The accuracy of the results has been demonstrated for aluminum and composite flat panels by comparing them against experimental tests and semi-analytical data, respectively. Since the good agreement, the FE method has been used to analyze the phenomena of dispersion, scattering, and mode conversion in aluminum and composite panels characterized by a structural discontinuity, as a stiffener. The research activity allowed emphasizing modes conversion at the stiffener, offering new observations with respect to state of the art. Converted modes propagate with a slightly slower speed than the incident ones. Reflected waves, instead, have been found to travel with the same velocity of the incident ones. Moreover, waves reflected in the composite stiffened plate appeared different from those that occurred in the aluminum one for the aspects herein discussed.

## 1. Introduction

A large amount of engineering infrastructure, aircraft, ground vehicles, ships and buildings have been ageing, becoming “structurally deficient” and in need of repairs, or “functionally obsolete” and in need of replacement [1]. Damage is a consequence of the operating and accidental loads that affect the structure during its in-service life and it must be tolerated from a design point of view to ensure the safety for users. Therefore, inspections and maintenance procedures are mandatory for “damage tolerant” structures, even though such procedures are highly costly and time demanding [2].

To design safer and more durable structures, the engineering community is aggressively pursuing novel sensing technologies and analytical methods that can be used to rapidly identify the onset and the evolution of damages in instrumented structural components, also defined as “smart structures”. Among various techniques, structural health monitoring (SHM) systems offer an automated solution to monitor the health of a structure by means of damage detection algorithms. SHM systems are developed through either the separate use or the integration of receiving sensors (passive method) and actuators (active method), opportunely equipped in structures [3]. Actually, the use of SHM systems is deeply associated with the damage tolerance design principles. Including SHM systems in the design phase could reduce the life-cycle cost, alleviating issues associated with regular inspections by replacing the scheduled maintenance with the as-needed one. This will consequently result in more specific and effective repairing operations.

Ultrasonic methods are successfully used for active SHM schemes [4]. Specifically, guided-wave (GW) testing has emerged considerably in the last decade as a prominent option for continuous and reliable monitoring [5]. Guided (Lamb) waves suit SHM intents because they allow monitoring of entire structures characterized also by geometrical discontinuities (e.g., stiffeners) with low power and costs. The diagnosis includes levels of excitation (easy and in a prescribed manner from a single location), detection, localization, and assessment of any detectable damage. In fact, GW propagation can be altered by the presence of damages/defects causing scattering and reflection phenomena together with attenuation of the signal amplitude [6]. Analyzing the output signals dataset and comparing it to a baseline one (recorded in a reference/pristine or previous state of the structure, and thus adopted as a benchmark) makes the structural diagnosis possible.

However, the propagation of Lamb waves is notoriously complex. Multiple wave modes ($S_{0}$, $A_{0}$, $S_{1}$, $A_{1}$, etc.) simultaneously exist, travelling with different speeds. Furthermore, the material inhomogeneity, the anisotropy and the multi-layered construction determine the dependence of wave modes on laminate layup, direction of wave propagation, frequency, and interface conditions. Finally, waves reflected from boundaries may easily conceal damage-scattered components in the signals [4]. So, to ensure precision, detection should be performed on a relatively small area.

The presence of structural details to improve the mechanical strength, such as splices or stiffeners, in primary and secondary components for many engineering structures (ship hulls, aircraft fuselages, etc.) further complicates the structural dynamics [7,8,9,10] and the understanding of wave propagation mechanisms. In particular, when encountering discontinuities, as for example stiffeners, complex phenomena can appear due to the multimodal and dispersive characteristics of Lamb waves: reflection, transmission, interference, mode conversion at the stringer [11], and attenuation [12]. Such phenomena may mask the presence of damages. The assessment of the dispersive characteristics can thus play a key role in setting up the SHM system parameters for the inspection phases, as the actuation signal frequency, so as to maximize the damage sensitivity also in stiffened components.

Dispersion equations solution can be achieved through several techniques [13,14,15]. Among all, a cost-effective approach is represented by the finite element (FE) method, which can significantly contribute to the prediction and the understanding of GW propagation mechanisms thanks to its ability to handle complex geometries and composite layups [16,17,18,19] while also considering environmental and operational conditions.

However, studies on the influence of the stiffener on propagation characteristics of GW signals and dealing with the definition of the dispersion curves or transmitted/converted/reflected modes are rarely reported. It has been demonstrated that the interaction of guided waves with stiffeners, such as ribs, stringers, or the integral stiffeners used in spacecraft structures, limits the size of the area over which SHM non-destructive evaluation systems can detect damage. An in-depth study of the interaction between GW signals and a structural discontinuity was performed by Han et al. in [20]. The results showed that the signal, undergoing a “T-shaped” transformation at the stiffener, generates various modes, among which the transmission signal accounted for the largest proportion. Ramadas et al. studied the interaction of the incident $A_{0}$ mode with a T-joint structural discontinuity in a composite structure through FE simulations and experiments [11]. Authors observed that when the $A_{0}$ mode interacts with the junction, a converted $S_{0}$ mode is generated.

Clearly, the presence of such “turning modes” could allow researchers to consider new inspection procedures in the discontinuity regions, based on conversion modes.

This work presents the analysis of guided wave propagation in aluminum and composite stiffened panels, addressed to a deep understanding of the wave propagation phenomenon in such complex structures. An extensive FE-based numerical investigation has been carried out to study the dispersion characteristics of the propagating guided waves in a SHM frequency range of interest for both aluminum and composite plates. In detail, for validation intents, dispersion curves in the flat panels have been extracted and compared with experimental results for the aluminum panel, and with analytical data provided by the dispersion calculator (Center of Lightweight Production Technology, German Aerospace Center (DLR), Augsburg, Germany) [21] for the composite one. As a result of the good level of accuracy, the stiffened panels have been numerically investigated in terms of modes conversion and dispersive behavior. Various converted/reflected wave modes in the signals have been effectively identified. It is observed that the presence of a spar in the structure significantly influences the incident modes, in terms of amplitude and propagation velocities, for transmitted and converted/reflected modes, respectively.

The remainder of the paper is organized as follows: In Section 2, GW propagation mechanisms in reinforced panels are detailed with reference to the actual state of the art. Section 3 presents the cases herein investigated: isotropic and composite panels, under both flat and stiffened configurations. Finally, results are discussed in Section 4, focusing on the dispersive behavior of GW propagating in flat plates and on GW conversion mechanisms around the structural discontinuity. In detail, dispersion curves of incident $S_{0}$ and $A_{0}$ modes are presented for isotropic and composite panels, under both flat and stiffened configurations, together with the dispersion curves of the converted/reflected modes. Section 5 concludes the paper.

## 2. Reflection, Attenuation, and Mode Conversion of Guided Waves in Stiffened Panels

Dispersion is the most significant characteristic of Lamb waves: the propagation velocity not only depends on the elastic constants and density of the material but also on excitation frequency and plate thickness [12].

Notoriously, multiple wave modes ($S_{0}$, $A_{0}$, $S_{1}$, $A_{1}$, etc.) simultaneously exist, travelling at different speeds. The two zero-order modes, the symmetric ($S_{0}$) and the antisymmetric ($A_{0}$) ones are, however, mainly used for damage detection in SHM applications.

Furthermore, when GWs propagate through defects/damages and/or structural/geometrical discontinuities, phenomena such as reflection, transmission, and modes conversion occur. In fact, as mentioned in Section 1, when GWs propagate in a stiffened panel, three types of waves can be observed at the reinforcement: transmitted, reflected, and those propagating along the reinforcement itself [20]. In this section, reflection, attenuation, and mode conversion phenomena are detailed with respect to the actual state of the art.

### 2.1. Reflection

When the wave interacts with the reinforcement, propagation mechanisms can become complex: the path followed by the scattered/reflected waves in composites is not well defined because of the anisotropic behavior of such materials.

For isotropic materials, the propagation direction of the reflected waves is governed by Snell’s law [22]:

(1) $$k_{i}\sin\theta_{i}=k_{r}\sin\theta_{r}$$

where $k_{i}$ and $\theta_{i}$ are the wavenumber and angle of the incident wave, respectively; $k_{r}$ and $\theta_{r}$ correspond to the wavenumber and the angle of the reflected wave, respectively. For an isotropic material it is possible to assume $\theta_{i}=\theta_{r}$: the wave is reflected from the structural discontinuity with the same characteristics of the incident wave. This means that considering a multi-modal wave, an incident S-type wave mode will be reflected at the spar interface, and the propagating mode along the reflected path will be sensed by the receiver as an S-type wave mode (no conversion). The same considerations apply for an incident A-type mode. For composites, Snell’s law can be considered still valid if the following strong hypotheses are assumed: the incident $A_{0}$ mode is reflected as $A_{0}$ only, and the $S_{0}$ mode is reflected as $S_{0}$ only [22].

Han et al. [20] proposed the multipath propagation model, that enabled the definition of different signals due to the presence of a geometrical discontinuity. In detail, authors focused on a T-shaped aluminum panel under both numerical and experimental points of view. The approach evaluates the propagation path of each echo to estimate its time of arrival, allowing separating of the direct signal, signals scattered from the stiffener, and signals reflected from the boundaries.

### 2.2. Attenuation

In previous studies related to GW propagation in stiffened plates, a substantial loss of the wave amplitude (~60%) was noted with respect to signals recorded in flat plates (no stiffener) [23,24]. Most of the energy is lost because when the incident wave reaches the reinforcement it decomposes into two components: one propagating up in the reinforcement, and one transmitting through the skin underneath the stiffener [23,24].

### 2.3. Mode Conversion

Han et al. [20] observed (through numerical analyses of Lamb wave propagation in an aluminum panel) that the interaction of the $A_{0}$ mode with the reinforcement generates reflected and transmitted $S_{0}$ modes, as mentioned in Section 2.1. This phenomenon depends on the frequency, on the geometry of the stiffener, and on its height.

Zheng et al. [25] investigated wave propagation in a stiffened composite panel excited with $S_{0}$ and $A_{0}$ modes simultaneously under 259 kHz excitation frequency. At first, the authors detected a reflected $S_{0}$ mode generated by the incident $S_{0}$ mode (indicated as SRS). Furthermore, modes conversion at the stiffener was detected: the incident $S_{0}$ mode produced a reflected $A_{0}$ mode (SRA), and the incident $A_{0}$ mode produced a reflected $S_{0}$ mode (ARS). The last detected mode was the reflected $A_{0}$ mode from the incident $A_{0}$ mode (ARA). Similarly, four transmission waves from the two incident modes at the stiffener were detected. Two transmitted modes derived from the incident $S_{0}$ mode (STS, STA) and two from the incident $A_{0}$ mode (ATS, ATA). In addition, reflected waves from the boundaries of the skin sheet were detected. However, the study was intended to investigate the damage detection and the mode conversion, but only under a specific frequency.

## 3. Cases of Study

In this work, the intent was to study the dispersive behavior of GWs in stiffened panels made of aluminum (isotropic) and composite materials. The dispersive behavior of 0-order modes and converted modes was investigated in the range of frequencies 50÷300 kHz. The calculation of dispersion curves for stiffened panels is very complex. In this regard, FE models have been used herein to better understand the propagation/conversion mechanisms at the stiffener interface. Very few papers have been found in literature dealing with GW propagation in stiffened panels, especially made of composite materials, and none of them deal with the dispersive behavior of converted/reflected modes. The dispersive behavior of such modes can help designers in improving the effectiveness and damaged sensitivity of GW based SHM systems. Moreover, with respect to reference [25], where converted modes were investigated only under a specific excitation frequency, 259 kHz, in this paper the range 50÷300 kHz has been considered.

In this section, the cases of study are detailed. Specifically, Section 3.1 is addressed to the description of the flat panels, while Section 3.2 deals with the stiffened panels.

Experimental tests have been performed only on the aluminum flat panel to define its dispersive behavior. Experimental data, in terms of dispersion curves, have been used to assess the level of accuracy of the proposed FE model in simulating GW propagation in isotropic flat panels. Concerning the composite flat panel, its dispersive behavior has been previously investigated through the dispersion calculator [21], widely recognized and used in literature. Analytical data provided by the calculator have been used to assess the reliability of the FE model in simulating GW propagation in composite flat panels by comparing again the dispersion curves. Subsequently, the modes conversion at the stiffener in the reinforced aluminum and composite panels has been investigated only numerically. Nevertheless, since the good agreement provided by the previous validation phase, predicted results can be considered accurate for these cases as well, according to the certification by analysis approach.

For all cases under investigation, numerical analyses have been performed by means of Abaqus^®^ CAE explicit code (Dassault Systems Simulia Corp, Providence, RI, USA), while an in-house Matlab^®^ (The MathWorks Inc., Natick, MA, USA) code has been used for the pre- and post-processing phases. The latter allows determining the time of flight (ToF) of the incident $S_{0}$ and $A_{0}$ modes (simultaneously activated), and of the converted modes, as widely explained in Section 4. Thus, once the distances between actuator/receivers and the ToF of the modes are known, it is possible to calculate the GW group velocities [16] and plot the dispersion curves.

### 3.1. Flat Panel

The first part of this work has been focused on the investigation of guided wave propagation in flat aluminum and composite plates.

**Figure 1:**
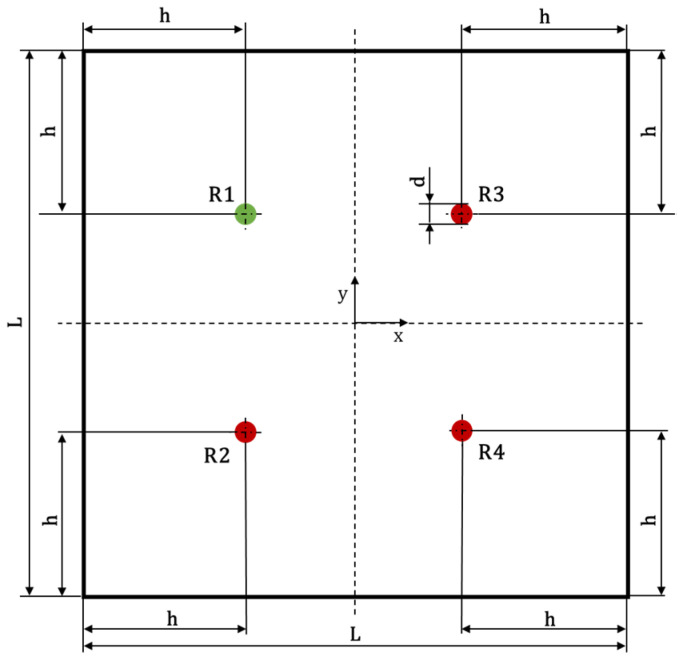
Panel geometry and sensors position.

The geometry of the plate is shown in Figure 1. Both aluminum and composite plates are characterized by a square shape and same in-plane dimensions (L=500 mm). The aluminum panel has a thickness of $t_{a}=2\;\mathrm{mm}$, while the composite one, made of CFRP (carbon fiber-reinforced polymer), is made up of 12 laminae for a total of $t_{c}=2.208\;\mathrm{mm}$ of thickness. The laminate stacking sequence is ${\left[45,-45,0,0,90,0\right]}_{s}$ and the 0° fiber direction is aligned with the x-axis of Figure 1. Aluminum and CFRP lamina mechanical properties are listed in Table 1.

A four Circular DuraAct (PI Ceramics) PIC255 piezoelectric transducers network has been used for both actuation and sensing of Lamb waves. The thickness and the radius of the PZT wafers are $t_{\mathrm{PZT}}=0.2\;\mathrm{mm}$ and $d_{\mathrm{PZT}}=10\;\mathrm{mm}$, respectively. The PZTs (indicated in Figure 1 as “R”), whose mechanical properties are listed in Table 1, have been surface mounted onto the specimen. They are located at a distance h=151 mm from the edges, Figure 1. Such transducers network has been used for the experimental tests carried out on the aluminum flat panel and numerically reproduced in all simulations related to flat panels. Additionally, material properties have been used to numerically characterize the modelled sensors.

In order to study the propagation and the dispersive behavior of Lamb waves at various frequencies, a chirp signal [26] has been used both experimentally and numerically. The transducer R1 has been chosen as actuator while the remaining PZTs have been used as sensing devices.

The chirp signal is given as follows:

(2) $$V_{\mathrm{chirp}}\left(t\right)=V_{\mathrm{in}}\left[H\left(t\right)-H\left(t-t_{\mathrm{chirp}}\right)\right]\sin\left(2\pi \left(f_{0}t+\frac{f_{1}-f_{0}}{t_{\mathrm{chirp}}}t^{2}\right)\right),$$

where $t_{\mathrm{chirp}}=0.25\;\mathrm{ms}$ is the duration of the chirp signal, $f_{0}\;$= 50 kHz is the start frequency, $f_{1}\;$= 300 kHz is the end frequency, $V_{\mathrm{in}}$ is the input amplitude, and H is the Heaviside function. The chirp signal allows users to achieve in a single test all dispersion curves in the selected frequency band. The tone-burst response, preferred due to the dispersive nature of Lamb waves [1], is then extracted by using the reconstruction procedure described in reference [27] to allow for the comparison for each frequency.

Experimentally, a 16 V peak-to-peak input amplitude was applied to the PZT actuator, using a TiePie waveform generator, and the TiePie digital oscilloscope was used to record the signals acquired at the PZT sensors with a sampling frequency of 2 MHz. The total recording duration of the experimental signals is $\mathrm{tot}=2\cdot 10^{-4}\;s$, and each measurement is recorded 32 times and averaged to improve the signal to noise ratio. The acquired signals from all four channels have a resolution of 12 bit. Each measurement was 0.2 ms long. All setup parameters have been reproduced in the simulations.

Concerning the numerical modelling, S4R conventional 2D shell elements have been used to model the panel, while C3D8R 3D solid elements have been chosen to model the PZTs. The plate and the sensors have been discretized with an average element size of 0.9 mm and 0.6 mm, respectively. These values allow discretizing 10 NPW (nodes per wavelength) at the $f_{1}$ carrier frequency, as reported in reference [12]. Details about the FE modelling can be found in Table 2.

To ensure the contact between sensors and plate, a node-to-surface contact formulation has been employed at the “tied” interfaces to simulate the adhesive layer between sensors and plate (not here modeled) [28].

Finally, the translational degrees of freedom of the four corners of the plate have been constrained as in the experiment, while, relative to the GW propagation, radial displacements equivalent to the input voltage of Equation (2) have been calculated through Equation (3), implemented in the in-house code pre-processing phase:

(3) $$d_{r}=R\frac{d_{31}}{h_{\mathrm{pzt}}}\left[\tilde{E}\frac{\left(1-\nu_{\mathrm{pzt}}\right)}{E_{\mathrm{pzt}}}\right]V_{\mathrm{chirp}},$$

where R, $E_{\mathrm{pzt}}$, $\nu_{\mathrm{pzt}}$, $d_{31}$, $h_{\mathrm{pzt}}$, and $V_{\mathrm{chirp}}$ are used as input parameters for the script. $\tilde{E}$ depends on geometry and mechanical properties of the investigated panel. This effective displacement has been applied on the upper actuator edge after having defined a proper polar coordinate system at the center of the actuator. Further details can be appropriately found in references [12,26].

### 3.2. Case Study: Stiffened Panels

**Figure 2:**
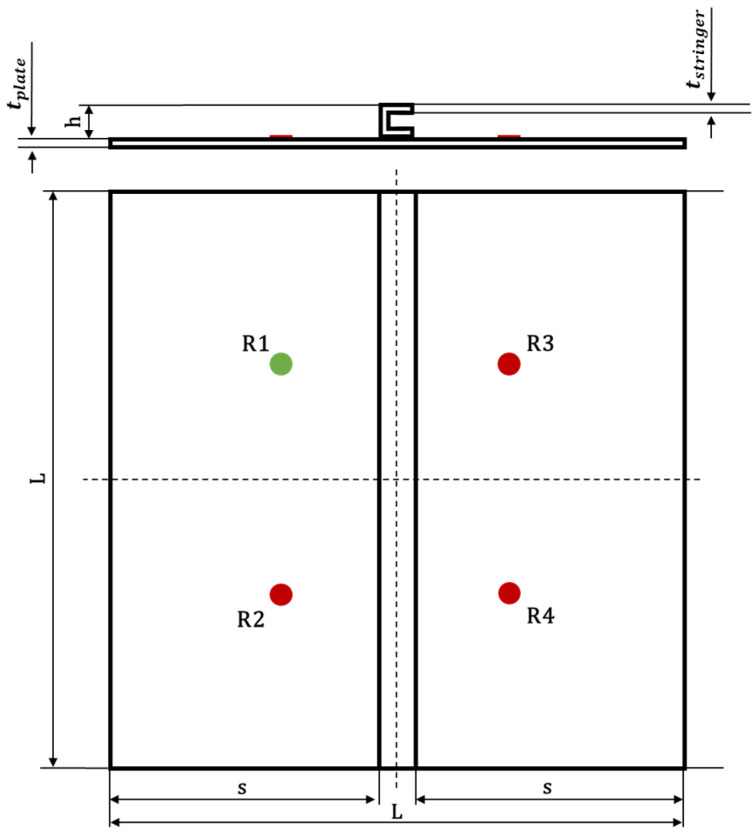
Stiffened panels geometry.

As mentioned in Section 1 and Section 2, stiffened panels have been studied only numerically. They have been modeled just equipping the flat panel with a C-cross-section stiffener, as shown in Figure 2, in order to study the influence of such reinforcement on GW propagation mechanisms. Specifically, the stringer has a height h=30 mm and a thickness $t_{\mathrm{stringer}}={\;t}_{\mathrm{plate}}$.

The geometrical discontinuity has been modelled with S4R type shell elements from the Abaqus^®^ Finite Elements library and it has been connected to the plate through tie constraints, allowing linking the degrees of freedom of the connected nodes.

For the geometry discretization, the sensors network layout, and for the boundary conditions, the same considerations as above apply. The developed FE model’s details for the stiffened panels are listed in Table 3.

**Figure 3:**
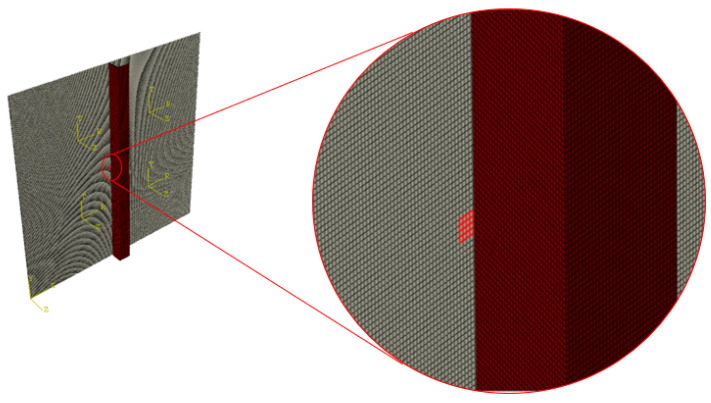
FE model of the stiffened panels and a focus on the elements set defined to evaluate the arrival instant of the wave in proximity of the stiffener.

In order to determine the ToF of a converted/reflected mode at the stiffener, a set of finite elements has been properly defined as visible in Figure 3. This way, it has been possible to record signals in proximity of the stiffener and use them in the post-process for the calculation of the dispersion curves of the converted/reflected waves. However, the procedure assumes that the reflected waves follow Snell’s law, as assumed in Section 2.

## 4. Results Analysis

Results for both aluminum and composite flat/stiffened panels have been herein discussed. The post process of the predicted signals has been addressed to highlight the effects of the stiffener on the propagation mechanisms, pointing the attention on the modes conversion.

Numerically, analyses have been performed under the explicit formulation of Abaqus^®^ code and the signals at the sensor positions have been calculated as the average of the in-plane strains, $\bar{\varepsilon }$, measured at all nodes defining each sensor. Similarly to Equation (3), based on piezoelectric relations and thanks to the code developed in Matlab^®^ environment, the signals in voltage can be calculated through the strain measurements as follows:

(4) $$V=Q_{s}\;\bar{\varepsilon },$$

where $Q_{s}$ is a conversion constant for the sensing (further mathematical details can be found in reference [12]).

Then, converted numerical signals and experimental ones have been processed by means of the developed code. In detail, their envelope has been evaluated and used to determine the ToFs of propagating, reflected, and transmitted waves. These envelopes have been then compared to those obtained from the flat plate to study the signals difference. Additionally, contour plots from the FE simulations have been used to detect the modes conversion and reflection phenomena.

### 4.1. Results for the Flat Panels

A chirp signal has been adopted for GWs excitation, as mentioned in Section 2. However, to concentrate most of the wave energy on a specific central frequency, recorded data have been reconstructed by means of a n-cycles sinusoidal tone burst Hanning windowed signal, with a step of 50 kHz (50:50:300 kHz). Once the distance between actuator-receivers and the ToF on all paths are known, it is possible to calculate the group velocity ($c_{g}$) of Lamb waves packets [16]. Considering that for an isotropic material $c_{g}$ does not depend on propagation direction, to compute the group velocity only the actuator 1–receiver 3 (R1–R3) path has been considered, whilst for the composite plate all paths have been investigated to highlight both the dispersion and slowness (dependence of the waves velocity on the direction of propagation) phenomena.

**Figure 4:**
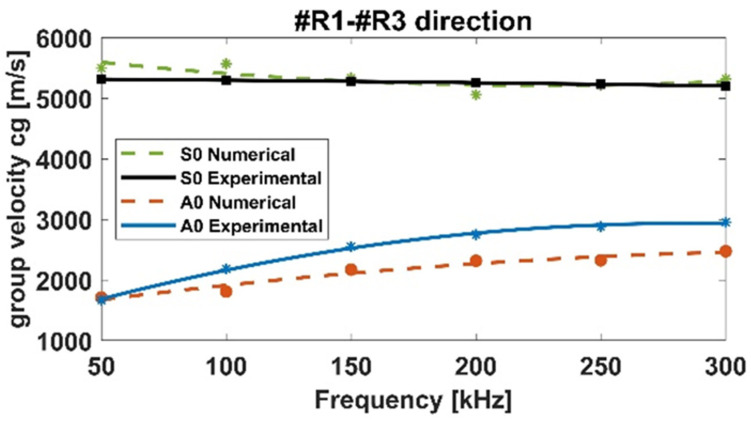
Dispersion curves for the flat aluminum panel.

**Figure 5:**
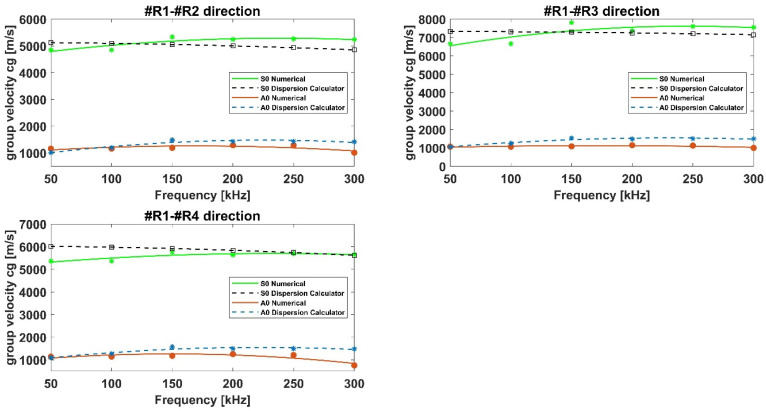
Dispersion curves for the flat composite panel.

Figure 4 and Figure 5 report the dispersion curves for the flat aluminum and composite panels, respectively. For the aluminum panel, numerical data have been compared against the experimental ones in terms of dispersion curves, Figure 4. A good agreement has been found, demonstrating the good modeling of the wave-propagation phenomenon. A slight but acceptable difference can be observed for $A_{0}$ mode curve. To improve the accuracy of the FE model, as widely demonstrated by authors in [29], 3D finite elements can be used to model the panel although affecting negatively the computational time.

With respect to the composite plate, Figure 5, the dispersion curves along different directions have been shown in order to highlight the effects of fibers orientation on wave propagation, whist for the isotropic panel the wave propagates with the same velocities omnidirectionally as expected. Numerical results in the selected frequency range have been compared to those obtained by means of the dispersion calculator. It is a Matlab^®^-based general-purpose tool that interactively allows users to create dispersion curves that simply define the material model and properties. It computes the phase and group velocity dispersion, as well as internal stress and displacement fields (mode shape) of Lamb and shear horizontal waves in isotropic and multilayered composites. The calculator is widely used and recognized in literature to calculate the dispersion curves in simple flat panel, also made of composite materials.

Again, according to Figure 5, numerical results appear in good agreement with dispersion calculator data for all paths. Specifically, it can be noticed that the $S_{0}$ mode group velocities for paths R1–R2 and R1–R4 are quite similar and higher for path R1–R3, due to the influence of 0° laminae (staking sequence: ${\left[45,-45,0,0,90,0\right]}_{s}$). Concerning the $A_{0}$ mode group velocities, they appear to be less sensitive to the laminae orientation. All these results can be considered accurate since they are in line with those available in literature [30,31] and presented by authors in reference [16]. Specifically, the modelling technique used herein to simulate GW propagation in aluminum and composite structures has been widely assessed by authors in previous research activities against analytical and experimental data [18,29].

### 4.2. Results for the Stiffened Panels

The aim of this section is to highlight the modes conversion in stiffened panels and, consequently, to define the dispersion curves of both incident and converted modes. Since one of the major problems in the identification of the converted modes concerns the signals interpretation, which is more complex in stiffened panels (stiffener-reflected waves overlap the waves reflected from the edges as well as the actuation signal), an in-depth signals analysis has been performed to point out the modes conversion mechanisms.

To better reveal the effects of the geometrical discontinuity on GW propagation mechanisms when the incident waves encounter it, a comparison between signals recorded in flat and stiffened configurations, in terms of both amplitude and mode conversion, has been mandatory. So, the difference between the envelopes of the signals recorded in the two analyzed configurations (flat plate and stiffened plate) has been studied to exclude all the reflections and scattering phenomena related to the edges of the plate. This way, attention has been paid only on the stringer-induced reflected and transmitted waves.

**Figure 6:**
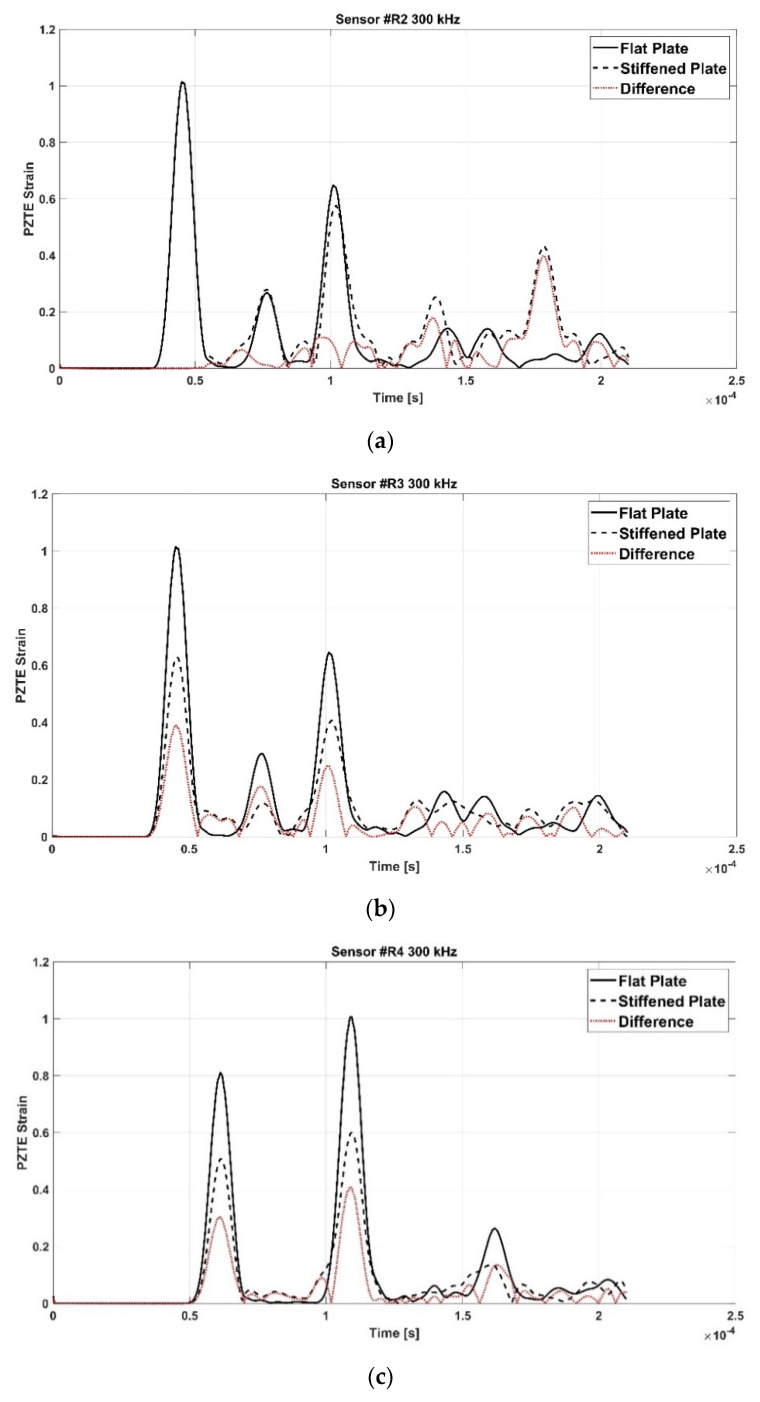
Flat (black solid line), stiffened (black dotted line), and difference (red solid line) signals in aluminum plate under 300 kHz carrier. (**a**) Sensor #R2 300 kHz; (**b**) Sensor #R3 300 kHz; (**c**) Sensor #R4 300 kHz.

Figure 6 shows the signals recorded in the flat configuration (solid line) and in the reinforced configuration (dotted line) of the aluminum panel, as well as the difference signal, for all the actuator R1-receiver Ri (i = 2,3,4) paths (a-b-c).

Such signals have been further extensively analyzed thanks to the support of the contour plots provided by the simulations. In fact, it has been possible, with a meticulous comparison and analysis of the simulation frames and of the signals, to identify the waves reflected by the stringer and those transmitted. S-type wave modes have been identified by activating in the contours the in-plane displacements only, while A-type wave modes by activating the through thickness displacements.

For the sake of brevity, only some examples are herein reported. According to the following figures, the phenomenon of the modes conversion mentioned in the previous sections is evident: $S_{0}$ mode waves incident to the stiffener generate reflected S-type waves (SRS) and reflected/converted A-type waves (SRA), transmitted S-type waves (STS) and transmitted/converted A-type waves (STA). In detail: SRS waves are reflected (symmetric) waves generated by the interaction (reflection) of the incident $S_{0}$ waves with the stiffener; SRA waves are converted (antisymmetric) waves generated by the reflection of the $S_{0}$ waves incident to the stiffener; STS waves correspond to a fraction of the incident $S_{0}$ mode propagating underneath the stiffener (transmitted); STA waves correspond to a fraction of the incident $S_{0}$ mode propagating underneath the stiffener (transmitted) and converted in antisymmetric waves. Therefore, according to the sensors position shown in Figure 2, SRS and SRA waves are captured only by sensor R2, while STS and STA waves are recorded only by sensors R3 and R4. Analogous behavior can be deduced for the incident $A_{0}$ waves. However, relatively to $A_{0}$ waves, only the transmitted A-type waves (ATA) have been identified. The other converted and reflected modes (ARS, ATS and ARA) have not been detected since their superimposition with boundary-reflected waves.

**Figure 7:**
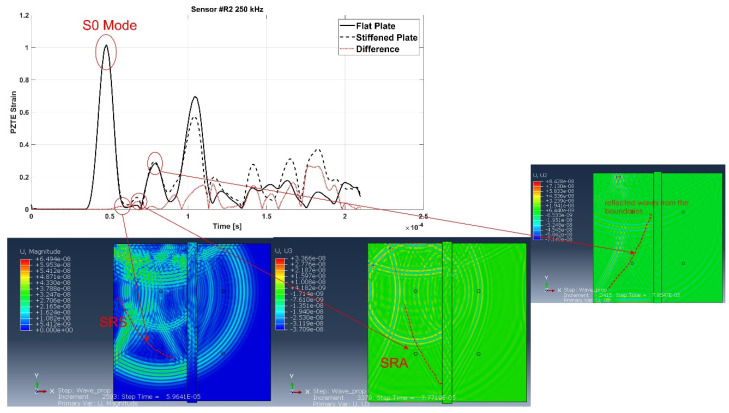
SRS and SRA modes at 250 kHz frequency detected by the R2 sensor—aluminum stiffened plate.

**Figure 8:**
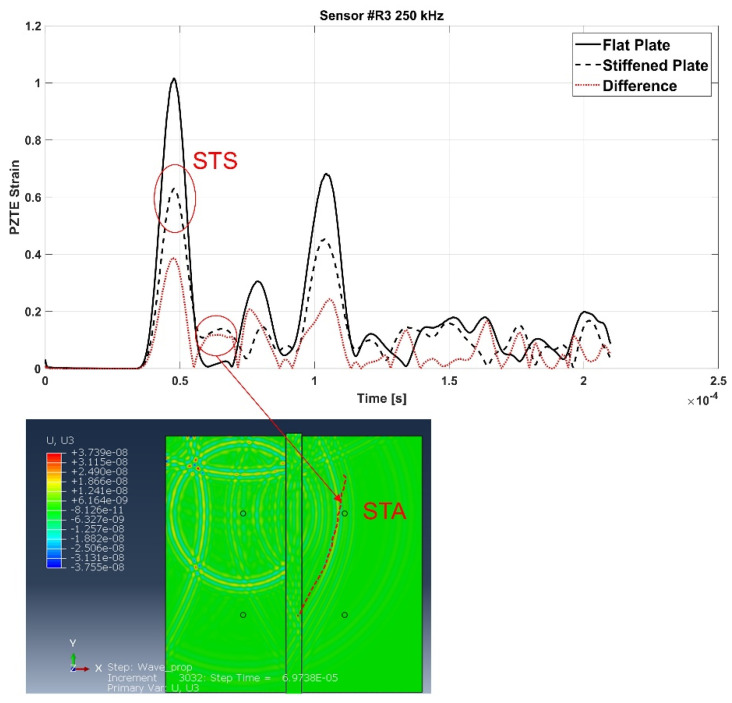
STA wave mode at 250 kHz carrier detected by the R2 sensor—aluminum stiffened plate.

**Figure 9:**
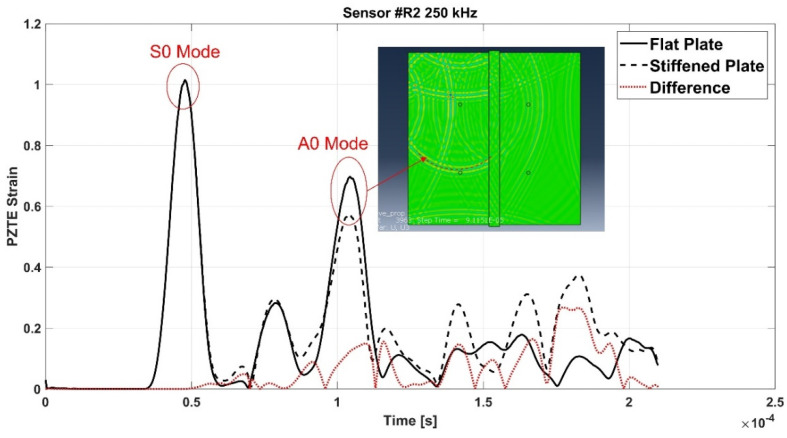
$A_{0}$ mode at 250 kHz carrier detected by the R2 sensor—aluminum stiffened plate.

**Figure 10:**
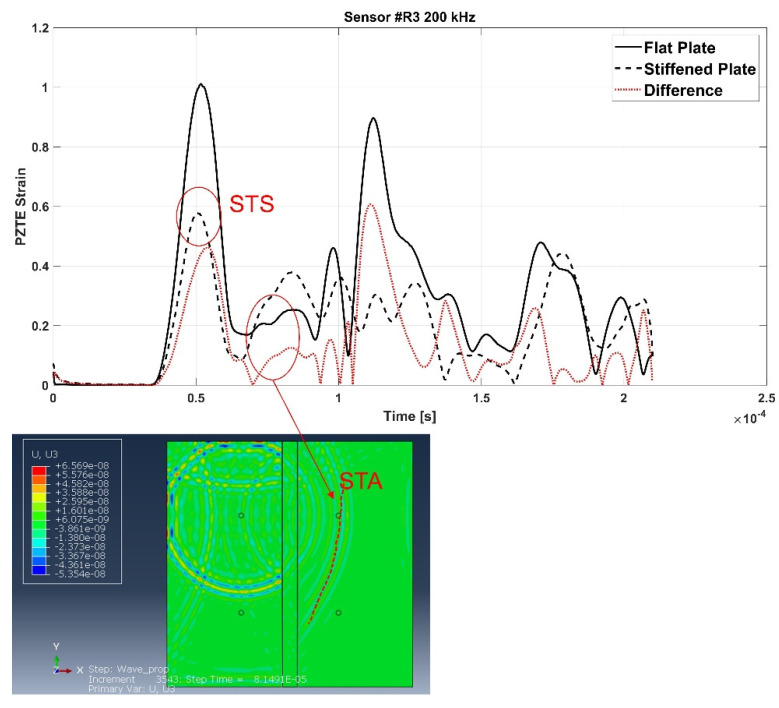
STA mode at 200 kHz carrier detected by the R3 sensor—aluminum stiffened plate.

In detail, for the aluminum test case, Figure 7 shows SRS and SRA wave modes at 250 kHz carrier detected by R2 sensor, while Figure 8 shows STA waves at 250 kHz carrier detected by R3 sensor. Figure 9 and Figure 10 show $A_{0}$ mode detected by sensor R2 at 250 kHz carrier and STA mode detected by sensor R3 at 200 kHz carrier.

In detail, according to the different signals shown in Figure 7 and Figure 9, it can be noted that the first packet at R2 sensor is null. This confirms that the $S_{0}$ mode travels without any disturbance to such sensor. Conversely, according to the difference signals shown Figure 8 and Figure 10, the presence of the first packet confirms that part of incident $S_{0}$ mode travels up in the reinforcement and part is transmitted through the skin underneath the stiffener.

So, once the SRS, SRA, STS, and STA waves at the various frequencies were identified, the group velocities have been calculated to construct the dispersion curves.

**Figure 11:**
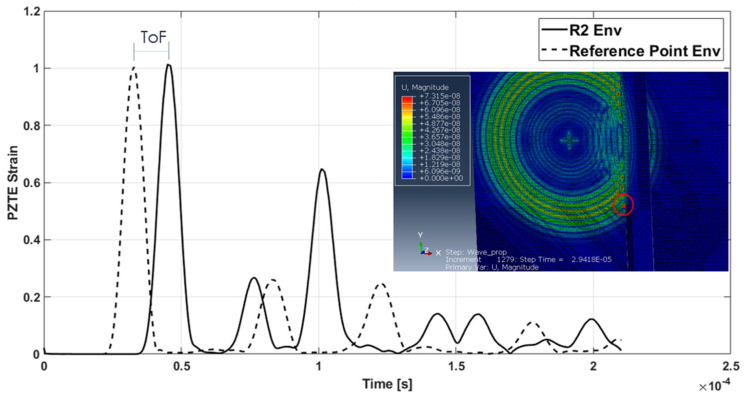
Waves ToF from reference point (red dot) to R2 sensor—aluminum stiffened plate.

Specifically, to evaluate the velocity of the converted/reflected waves, it has been assumed that these are born at the instant in which the incident wave reaches the stringer. This time instant has been evaluated by analyzing signals recorded at the elements set highlighted in Figure 3. The time between the peaks of the incident wave and of the reflected wave detected by R2 sensor is the time of flight (Figure 11) used for the reflected waves velocity calculation.

**Figure 12:**
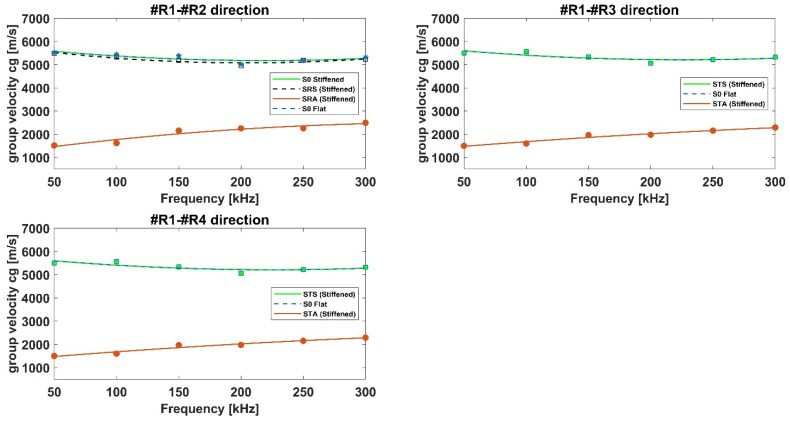
Comparison of the group velocities of the incident and converted/reflected $S_{0}$ modes detected in the flat and reinforced configurations – aluminum panels.

The dispersion curves for the reinforced aluminum panel have been constructed and compared to those related to the flat configuration. Figure 12 reports the comparison of the group velocities of the incident and converted/reflected $S_{0}$ mode detected in the flat and reinforced configurations for the aluminum panels. In detail, with respect to the R1–R2 path, no relevant changes in ToFs can be identified between the $S_{0}$ mode recorded at both flat and stiffened configurations. This is because no disturbance affects such path. In addition, for the stiffened panel, it can be noticed that, caused by the interaction between the incident $S_{0}$ mode and the stiffener, a reflected symmetric mode (SRS) is generated at the stiffener interface, reaching R2 receiver with a group velocity quite close to the incident $S_{0}$ mode. At the same time, due to the same interaction between the $S_{0}$ mode and the stiffener, part of such mode converts in an antisymmetric one (SRA), characterized by a slower group velocity.

Paths R1–R3 and R1–R4 reveal similar characteristics. The symmetric mode STS, representing the part of the incident $S_{0}$ mode travelling underneath the stiffener, travels with the same velocity of $S_{0}$ mode recorded in the flat configuration. Hence, the main difference between the flat and stiffened configurations concerns the amplitude of the signals, as visible in Figure 10. Regarding the STA mode, it is found to travel significantly slower than the incident $S_{0}$ mode but with the same velocity of the SRA mode.

**Figure 13:**
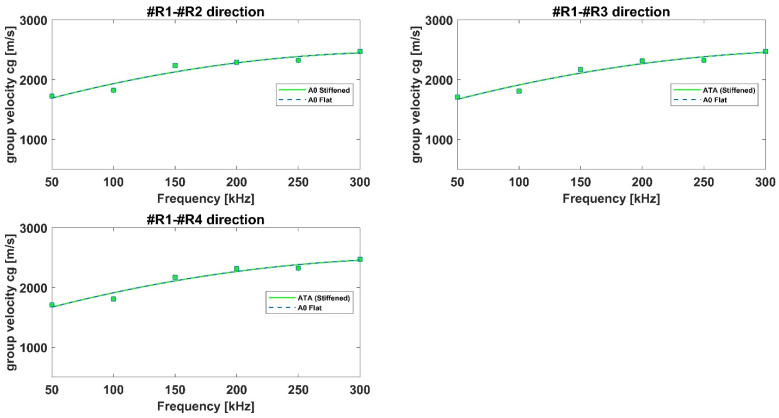
Comparison of the group velocities of the incident and converted/reflected $A_{0}$ modes detected in the flat and reinforced configurations – aluminum panels.

Figure 13 reports the comparison of the group velocities of incident $A_{0}$ and transmitted ATA modes detected in the flat and reinforced configurations of the aluminum panels.

According to Figure 13, relative to the $A_{0}$ mode travelling in the flat and stiffened configurations recorded at receiver R2, no differences in ToFs have been found. At receivers R3 and R4, the $A_{0}$ mode travelling underneath the stiffener (ATA mode) has been found to travel with the same group velocity of $A_{0}$ mode in the flat configuration.

In other words, analyzing Figure 12 and Figure 13, it can be clearly seen that SRA, STA, ATA (stiffened panel) and $A_{0}$ (flat panel) modes travel with quite similar velocities.

As aforementioned, due to the finite dimensions of the plates, it has not been possible to analyze other modes (ARS, ATS and ARA). Such modes in fact cannot be easily detected due to their overlapping with boundary-reflected waves.

**Figure 14:**
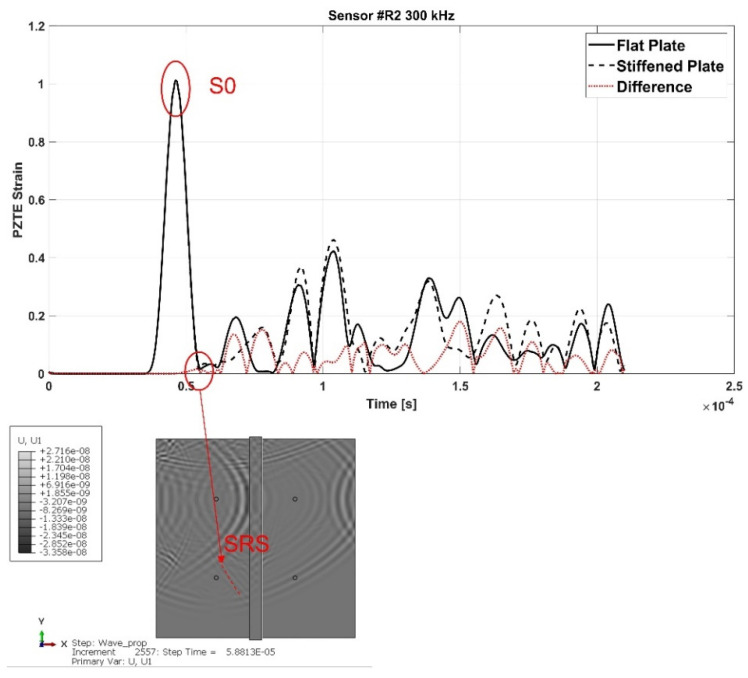
SRS mode at 300 kHz carrier detected by the R2 sensor – composite stiffened panel.

**Figure 15:**
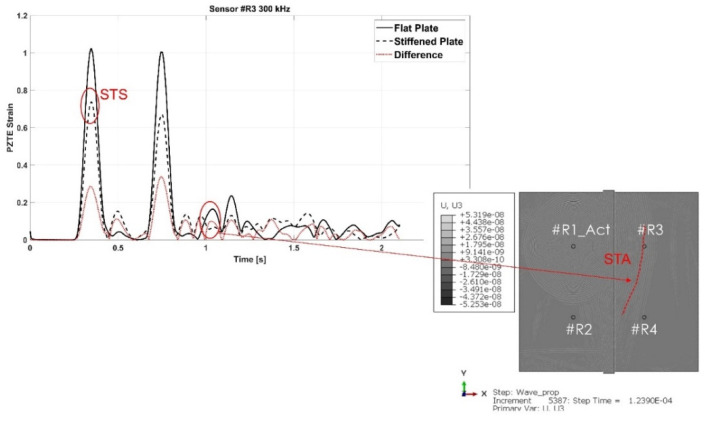
STA mode at 300 kHz carrier detected by the R3 sensor—composite stiffened panel.

As for the aluminum stiffened plate, Lamb waves propagation mechanisms, in terms of group velocities, have been analyzed in the composite stiffened panel and compared to the flat configuration. Again, an accurate analysis of the collected data and of the contour plots has been necessary to properly detect the converted/reflected modes and their propagation mechanisms. Similar considerations about the conversion/reflection mechanisms as for the aluminum panel apply here. As an example, Figure 14 shows the SRS mode reflected by the stiffener and intercepted by the sensor R2, while Figure 15 highlights the STA mode detected at sensors R3.

**Figure 16:**
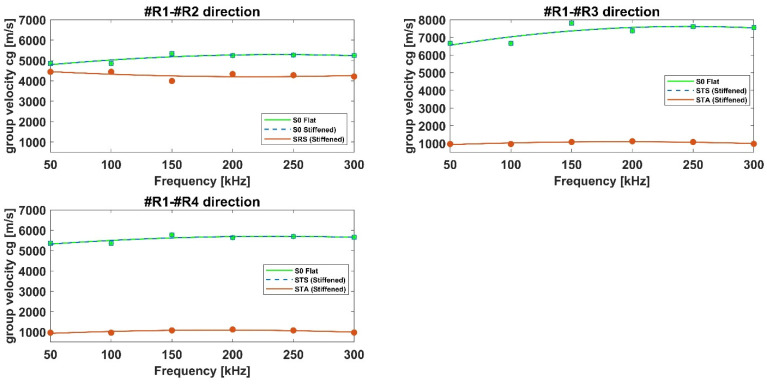
Comparison of the group velocities of the incident and converted/reflected $S_{0}$ modes detected in the flat and reinforced configurations —composite panels.

**Figure 17:**
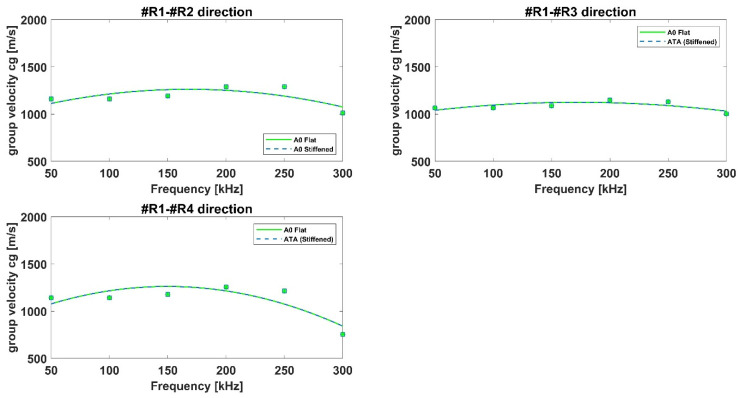
Comparison of the group velocities of the incident and converted/reflected $A_{0}$ modes detected in the flat and reinforced configurations—composite panels.

Then, the SRS, STS, ATA, and STA modes’ dispersion curves have been constructed, as shown in Figure 16 and Figure 17. As for the aluminum panels, similar considerations can be drawn. The main difference between the aluminum and composite panels is that for the composite stiffened one it has not been possible to identify the SRA waves as their amplitude is negligible compared to the rest of the signal. Therefore, only the S-type waves reflected by the stringer (SRS), and the A-type transmitted waves (STA) have been identified.

Moreover, while in the aluminum panel SRS mode has been found to travel with a group velocity quite equals to the incident $S_{0}$ mode, the same cannot be stated for the composite one. In fact, according to Figure 16, it can be noticed that the transmitted STS mode travels with the same velocity as the $S_{0}$ mode recorded in the flat plate, whilst the SRS mode travels slower than the incident $S_{0}$ mode. This latter effect can be observed by the dispersion curves recorded at sensor R2.

Figure 17 reports the comparison of the group velocities of the incident $A_{0}$ and transmitted ATA modes detected in the flat and reinforced configurations for the composite panels. Analyzing Figure 16 and Figure 17, it can be clearly noticed that STA, ATA (stiffened panel), and $A_{0}$ (flat panel) modes travel with different velocities: ATA and $A_{0}$ modes propagation is almost similar whilst the STA mode is slower.

In general, for both aluminum and composite panels, converted modes have been found to travel slower than the incident, transmitted, and reflected modes.

## 5. Conclusions

The aim of this work is to propose a numerical methodology based on FE method to investigate the dispersive behavior of guided waves converted and reflected modes in reinforced aluminum and composite structures, highlighting their differences. The dispersion curves of such modes can help designers in improving the damage detection sensitivity of Lamb waves based SHM systems.

Most of the papers presented in literature, in fact, deal with simple cases of study and dispersion curves for reinforced panels are not provided. Hence, an extensive numerical investigation has been herein presented. For the purpose, four different FE models have been developed consisting of: (i) an aluminum flat plate; (ii) a composite flat plate; (iii) an aluminum stiffened plate and (iv) a composite stiffened plate. To verify the reliability of the proposed FE modelling technique, the construction of the dispersion curves has been firstly carried out for simpler cases of study (flat plates) and validated against experimental data for the aluminum panel, and against analytical data, provided by the dispersion calculator, for the composite panel. Differently, for the stiffened panels, given the good level of accuracy of the results, also in line with previous authors’ research activities dealing with complex real structures, the former validation phase can be used under a certification by analysis approach. So, the FE modelling technique for GW propagation can also be used to investigate more complex phenomena as the ones related to the material anisotropy as well as the ones arising from the interaction with geometrical discontinuities.

In this work, modes conversion in both composite and aluminum stiffened panels have been compared. In particular, for the stiffened panels, the SRS, SRA, STS, and STA dispersion curves have been constructed. It has been observed that converted modes propagate with a slightly slower speed than the incident $S_{0}$ one. This phenomenon could be due to the loss of energy caused by the presence of the stiffener.

For the reinforced aluminum panel, caused by the interaction between the incident $S_{0}$ mode and the stiffener, a reflected symmetric mode (SRS) is generated at the stiffener, reaching the R2 receiver with a group velocity quite close to the incident $S_{0}$ mode. Conversely, for the composite panel, SRS mode travels slower than the incident $S_{0}$ mode. In general, for both aluminum and composite panels, the incident $S_{0}$ mode transmitted underneath the stiffener (STS), has been found to travel with the same velocity as the $S_{0}$ mode recorded in the flat configurations, but with different (lower) amplitude. Furthermore, for aluminum, SRA, STA, ATA (stiffened panel), and $A_{0}$ (flat panel) modes travel with quite similar velocities. Instead, for the composite stiffened panel, it has not been possible to identify the SRA waves as their amplitude is negligible compared to the rest of the signal, while ATA (stiffened panel) and $A_{0}$ (flat panel) modes propagation is almost similar and the STA mode is slower.

## Figures and Tables

**Table 1:** Material properties of Al 6061 plate, CFRP composite lamina, and PIC255 sensors.

Material Properties	Symbol	Units	Al 6061	CFRP Lamina	PZT
Mass density	ρ	$\left[{\mathrm{kg}\;m}^{-3}\;\right]$	2700	1600	7850
Young’ s modulus	E	[GPa]	69	−	76
Longitudinal Young’s modulus	$E_{11}$	[GPa]		140.2	−
Transversal Young’s modulus	$E_{22}$	[GPa]		8.675	−
Transversal Young’s modulus	$E_{33}$	[GPa]		8.675	−
Shear modulus	G	[GPa]	26	−	29
Shear modulus	$G_{12}$	[GPa]		4.29	−
Shear modulus	$G_{13}$	[GPa]		4.29	−
Shear modulus	$G_{23}$	[GPa]		3.1	−
Poisson’s ratio	ν	−	0.33	−	0.32
Poisson’s ratio	$\nu_{12}$	−		0.312	−
Poisson’s ratio	$\nu_{13}$	−		0.312	−
Poisson’s ratio	$\nu_{23}$	−		0.4	−
Dielectric constant	$K_{3}$	−		−	1280
Piezoelectric charge constant	$d_{31}$	$\left[\;10^{-9}{\;\mathrm{mmV}}^{-1}\right]$		−	−180

**Table 2:** FE modelling details for the flat panels.

Approach	Part	Element Type	Elements Number	Nodes Number	DoF (Degrees of Freedom)
3D Solid	PZTs	C3D8R	2176	3588	10,764
2D Shell	Plate	S4R	309,136	310,249	1,861,494

**Table 3:** FE modelling details for the stiffened panels.

Approach	Part	Element Type	Element Number	Nodes Number	DoF
3D Solid	PZTs	C3D8R	2176	3588	10,764
2D Shell	Plate	S4R	309,136	310,249	1,861,494
2D Shell	Stiffener	S4R	55,044	55,700	334,200

## Data Availability

Not applicable.
